# SMARCA2-deficiency confers sensitivity to targeted inhibition of SMARCA4 in esophageal squamous cell carcinoma cell lines

**DOI:** 10.1038/s41598-019-48152-x

**Published:** 2019-08-12

**Authors:** Katharina Ehrenhöfer-Wölfer, Teresa Puchner, Cornelia Schwarz, Janine Rippka, Silvia Blaha-Ostermann, Ursula Strobl, Alexandra Hörmann, Gerd Bader, Stefan Kornigg, Stephan Zahn, Wolfgang Sommergruber, Norbert Schweifer, Thomas Zichner, Andreas Schlattl, Ralph A. Neumüller, Junwei Shi, Christopher R. Vakoc, Manfred Kögl, Mark Petronczki, Norbert Kraut, Mark A. Pearson, Simon Wöhrle

**Affiliations:** 1 Boehringer Ingelheim RCV GmbH & Co KG, 1120 Vienna, Austria; 2 Department of Cancer Biology, University of Pennsylvania, Philadelphia, PA 19104 USA; 3 Cold Spring Harbor Laboratory, Cold Spring Harbor, NY 11724 USA

**Keywords:** Targeted therapies, Small molecules

## Abstract

SMARCA4/BRG1 and SMARCA2/BRM, the two mutually exclusive catalytic subunits of the BAF complex, display a well-established synthetic lethal relationship in SMARCA4-deficient cancers. Using CRISPR-Cas9 screening, we identify SMARCA4 as a novel dependency in SMARCA2-deficient esophageal squamous cell carcinoma (ESCC) models, reciprocal to the known synthetic lethal interaction. Restoration of SMARCA2 expression alleviates the dependency on SMARCA4, while engineered loss of SMARCA2 renders ESCC models vulnerable to concomitant depletion of SMARCA4. Dependency on SMARCA4 is linked to its ATPase activity, but not to bromodomain function. We highlight the relevance of SMARCA4 as a drug target in esophageal cancer using an engineered ESCC cell model harboring a SMARCA4 allele amenable to targeted proteolysis and identify SMARCA4-dependent cell models with low or absent SMARCA2 expression from additional tumor types. These findings expand the concept of SMARCA2/SMARCA4 paralog dependency and suggest that pharmacological inhibition of SMARCA4 represents a novel therapeutic opportunity for SMARCA2-deficient cancers.

## Introduction

Synthetic lethality is a well-established concept developed from genetic studies in fruit flies and yeast^1–5^ where concomitant deficiency of two (or more) synthetic lethal partner genes is lethal, while individual loss-of-function of any of the synthetic lethal partners is compatible with cell viability. Selective targeting of tumors harboring loss-of-function alterations of a synthetic lethal gene by pharmacological inhibition of the functional synthetic lethal partner has been proposed for cancer therapy. The response of patients with BRCA1/BRCA2-deficient tumors to Polymerase (PARP) inhibitors has demonstrated how this approach can be successfully exploited in the clinic^6,7^. Several other synthetic lethal interactions highlighting potential cancer drug targets have been described in pre-clinical models^7–9^. Most notably, synthetic lethal interactions of paralog genes, such as *ENO1/ENO2* or *STAG1/STAG2*^10–12^ with redundant - but cell essential - functions result in acute and strong dependencies on the functional paralog in tumor cells deficient for the corresponding paralog gene.

Cancer-relevant paralog dependencies are prominently observed for BRG1-Associated Factor (BAF) chromatin remodeling complexes^13^. BAF complexes, also referred to as Switch/Sucrose Non-Fermenting (SWI/SNF) complexes, are large, multi-subunit complexes that catalyze chromatin remodeling via ATP-dependent mobilization of nucleosomes, thereby impacting on chromatin accessibility of the transcriptional machinery and global gene expression^14^. The core BAF complex consists of a DNA-dependent ATPase, as the sole known enzymatic activity, and three subunits (SMARCB1/BAF47/SNF5, SMARCC1/BAF155 and SMARCC2/BAF170) that are critical for structural integrity and chromatin recruitment. BAF complexes are further variably composed of accessory components in a cell-type and context specific manner^15^. Multiple BAF components are represented by paralogous subunits that are incorporated into the complex in a mutual exclusive fashion. Most notably, the BAF ATPase function may be exerted by either SMARCA4 or its homologous paralog SMARCA2^16,17^.

Loss-of-function alterations of individual BAF complex components, such as bi-allelic inactivation of the BAF core component SMARCB1 in malignant rhabdoid tumors^18,19^, are known oncogenic driver events, indicating a tumor suppressive role for BAF complexes^20^. Cancer-associated BAF alterations do not result in complete abrogation of BAF activity, which would be detrimental for viability of most cells, implying that tumors are dependent on the aberrant function of the residual BAF complex^13^. This is exemplified by the ATPase subunit SMARCA4, which is frequently inactivated by loss-of-function mutations or epigenetic silencing in non-small cell lung cancer (NSCLC) and other tumor types^21–24^. Consequently, SMARCA4-deficient cell models are rendered highly dependent on the paralog ATPase SMARCA2 to maintain residual BAF activity^25–27^.

Esophageal carcinoma, which is histologically classified as adenocarcinoma or squamous cell carcinoma^28^, is one of the most common cancers worldwide with a five-year-survival rate ranging from 15 to 25%^29^. Esophageal squamous cell carcinoma (ESCC) is the predominant subtype of esophageal carcinoma, with particular high incidence in Eastern and Central Asia^30,31^. Treatment of ESCC is currently largely limited to surgery and chemotherapy/radiotherapy approaches^32^. With the exception of a few actionable genomic alterations, such as *EGFR* amplification, targeted therapy options for ESCC are limited^33–36^.

In this study, using a pooled single guide RNA (sgRNA) viability screen targeting epigenetic factors in a panel of ESCC cell models, we identify the BAF chromatin remodeling ATPase subunit SMARCA4 as a novel dependency in a subset of ESCC cell lines with low or absent expression of SMARCA2. By applying SMARCA2 reconstitution and knock-out strategies in ESCC cell models, we define a novel synthetic lethal relationship reciprocal to the SMARCA2 dependency observed in SMARCA4-deficient cancer cells. This concept is further extended to non-ESCC cancer cell models, indicating SMARCA4 as a potential therapeutic target in tumors with low or absent expression of SMARCA2.

## Results

### CRISPR-Cas9 screening identifies SMARCA4 as a novel dependency in ESCC

**Figure 1:**
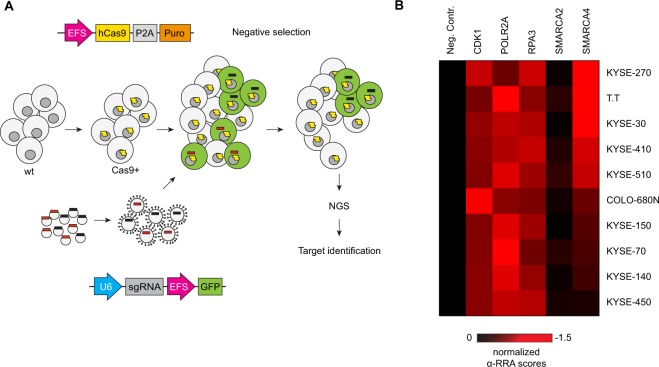
SMARCA4 is a dependency in a subset of ESCC cell models. (**A**) Scheme of CRISPR-Cas9 depletion screens. Library plasmids were amplified and lentivirally packaged. The vectors encode for the respective sgRNA and GFP. Stably Cas9 expressing cells were transduced with the pooled library (using a multiplicity of infection of approximately 0.3) and cultured for 18 population doublings. sgRNA read counts from the genomic DNA obtained at the end of the screen were determined by next-generation sequencing (NGS) and compared to read counts derived from the DNA plasmid library. Sequences of sgRNAs from the library are listed in Supplementary Dataset File 1. (**B**) Heatmap of epigenome-wide CRISPR-Cas9 depletion screens in ten ESCC cell lines. Data shown are α-RRA scores (robust ranking aggregation) from pooled sgRNA depletion screens relative to mean of the α-RRA scores of three essential genes (*CDK1*, *POLR2A*, *RPA3*). The mean of the positive controls was set to −1. Cell lines are ranked by SMARCA4 α-RRA scores. α-RRA scores for all genes included in the screen and raw depletion values (provided as log_2_ fold-change values) for single gRNAs are listed in Supplementary Tables 3 and 4.

To identify potential novel drug targets in ESCC, we applied domain-directed CRISPR-Cas9 screens^37^ across ten ESCC cell lines. Stable Cas9-expressing cell lines were transduced with a sgRNA library consisting of >1300 sgRNAs targeting 179 epigenetic regulators^38^ and negative selection of sgRNA-expressing cells was monitored by next-generation sequencing after 18 population doublings (Fig. 1A). From these screens, we identified SMARCA4 as a strong dependency in a subset of the ESCC cell lines (Fig. 1B, Supplementary Dataset File 2 and 3). Six ESCC models (KYSE-270, T.T, KYSE-30, KYSE-410, KSYE-510 and COLO-680N) displayed relative Robust Rank Aggregation (α-RRA) scores below −0.5 similar to the known essential genes *CDK1*, *POLR2A* and *RPA3*. In contrast, four cell lines (KYSE-150, KYSE-70, KYSE-140 and KYSE-450) showed no or less dependency (α-RRA scores ≥ −0.5) on SMARCA4. Of note, none of the ESCC lines showed dependency on the paralogous BAF subunit SMARCA2 (Fig. 1B).

Reciprocal to the known dependency of SMARCA4-deficient cancer cell lines on SMARCA2^25–27^, the majority of SMARCA4-dependent ESCC cell lines (KYSE-270, KYSE-30, KSYE-510 and COLO-680N) displayed low or non-detectable levels of SMARCA2 mRNA and protein expression (Supplementary Fig. S1A). In contrast, T.T and KYSE-410 cells were dependent on SMARCA4 despite readily detectable SMARCA2 expression. SMARCA4 mRNA and protein expression was detected in all ESCC lines (Supplementary Fig. S1A).

To establish a link between SMARCA2 expression levels and dependency on SMARCA4 beyond the cell models used in this study, we analyzed functional genomic datasets from recent pooled shRNA and CRISPR-Cas9 viability screens^39,40^, including 14 and 16 ESCC cell lines, respectively. In both datasets, sensitivity to SMARCA4 loss-of-function was anti-correlated with SMARCA2 expression levels (Supplementary Fig. S1B).

We performed depletion studies using individual sgRNAs to corroborate and strengthen the findings of the pooled CRISPR-Cas9 screens (Supplementary Fig. S2A). A higher frequency of loss-of-function mutations are observed with sgRNAs directed to sequences encoding functionally relevant protein domains^37^. Therefore, 17 different sgRNAs targeting DEXDc-, helicaseC-, bromodomain or N-terminal coding regions of SMARCA4 were tested in the KYSE-30 and KYSE-510 cell lines (Supplementary Fig. S2B). Both cell lines showed strong depletion effects with sgRNAs targeting the ATPase encoding regions, while efficacious depletion with bromodomain-directed sgRNAs was observed in KYSE-30 cells only. In contrast, N-terminal targeting sgRNAs displayed only mild effects in both models (Supplementary Fig. S2B).

**Figure 2:**
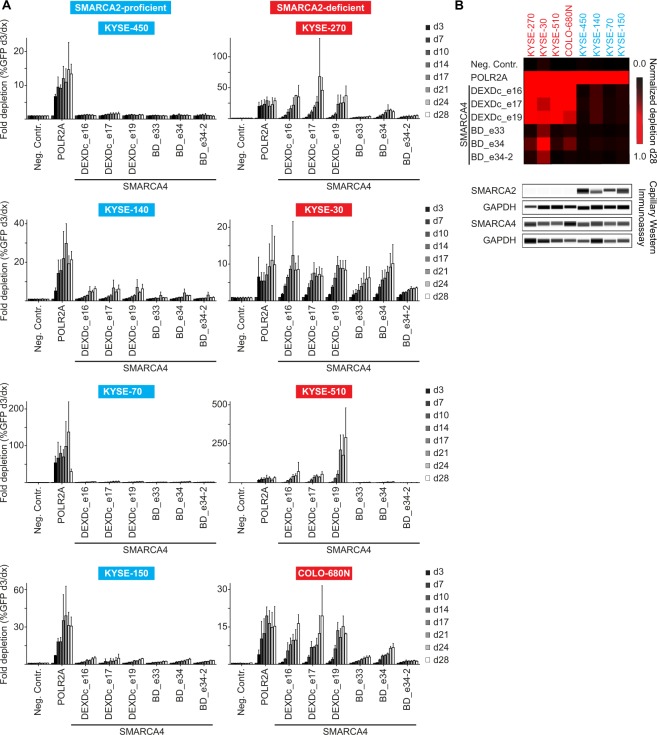
Selective dependency of SMARCA2-deficient ESCC models on SMARCA4. (**A**) Time-resolved CRISPR-Cas9 depletion studies. Fold depletion of sgRNA- and GFP-co-expressing cells relative to day 3 (d3) post-transduction was assessed by flow cytometry analysis in SMARCA2-proficient (blue) and SMARCA2-deficient (red) cell lines over 28 days. Three individual sgRNAs were directed against the DEAD-like helicase superfamily (DEXDc) domain and the bromodomain (BD), respectively. Non-targeting (Neg. Contr.) and cell essential gene (POLR2A) targeting sgRNAs were included as negative and positive controls. Data are represented as mean ± SD of three independent experiments. (**B**) Heatmap summary of studies shown in (A) normalized to depletion of POLR2A-directed sgRNAs at day 28 (d28) and capillary Western immunoassay for SMARCA2 and SMARCA4 of ESCC cell lines shown in (A). GAPDH expression was used to monitor equal loading.

We then screened a selected panel of SMARCA2-proficient and -deficient ESCC lines in a time-resolved fashion using the three most efficacious DEXDc- and bromodomain targeting sgRNAs (Fig. 2A). Absence of SMARCA2 protein expression in KYSE-270, KYSE-30, KYSE-510 and COLO-680N cells was confirmed by capillary Western immunoassay (Fig. 2B). No strong depletion effects were observed upon knock-out of SMARCA4 in the SMARCA2-proficient cell models KYSE-450, KYSE-140, KYSE-70 and KYSE-150 over a course of 28 days. In contrast, targeting SMARCA4 in the SMARCA2-deficient cell models KYSE-270, KYSE-30, KYSE-510 and COLO-680N resulted in depletion effects similar or stronger than observed for the POLR2A positive control sgRNA (Fig. 2A). With the exception of KYSE-30, DEXDc-targeted sgRNAs showed stronger and faster depletion than bromodomain-directed sgRNAs in SMARCA2-deficient cells (Fig. 2A,B), indicating a requirement of SMARCA4 ATPase activity in the context of SMARCA4 dependence^37^. Of note, SMARCA4 bromodomain-targeting, but not ATPase-directed, sgRNAs were included in the pooled sgRNA library. The effect observed with SMARCA4 bromodomain-targeting sgRNAs in the individual sgRNA depletion studies (Fig. 2) thus might be exacerbated in the pooled negative selection setting (Fig. 1B).

These results highlight the BAF subunit SMARCA4 as a novel vulnerability in ESCC characterized by low or absent expression of its homologous ATPase SMARCA2.

### SMARCA4 dependency is linked to its ATPase function

**Figure 3:**
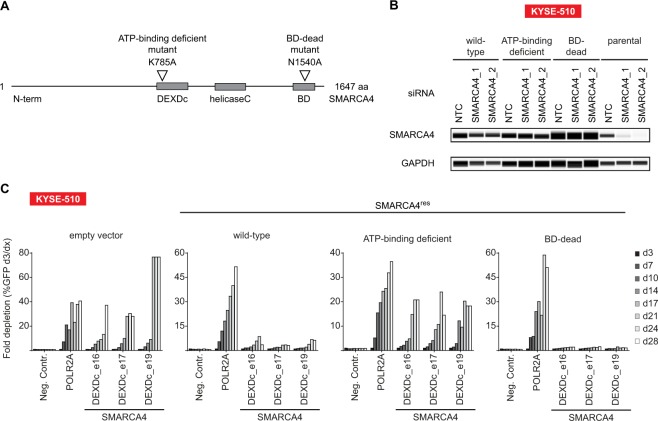
SMARCA4 dependency in SMARCA2-deficient ESCC cell lines is linked to its ATPase function. (**A**) Schematic representation of SMARCA4 domain structure. Location of ATP-binding- and bromodomain (BD)-inactivating mutations in siRNA/sgRNA-resistant SMARCA4 (SMARCA4^res^) is indicated. DEXDc, DEAD-like helicases superfamily; BD, bromodomain. (**B**) SMARCA2-deficient KYSE-510 cells were stably transduced with wild-type or mutant forms of SMARCA4^res^. Transgene expression was monitored by capillary Western immunoassay in presence of siRNA-mediated knockdown of endogenous SMARCA4. Lysates were prepared 72 h post siRNA transfection. GAPDH expression was used to monitor equal loading. (**C**) Time-resolved CRISPR-Cas9 depletion studies in SMARCA4^res^ expressing KYSE-510 cells. Fold depletion of sgRNA- and GFP-co-expressing cells relative to day 3 (d3) post-transduction was assessed by flow cytometry analysis over 28 days (n = 1 experimental replicate).

The requirement of SMARCA4 ATPase and bromodomain function in SMARCA2-deficient ESCC cells was further assessed by generating siRNA/sgRNA-resistant SMARCA4 (SMARCA4^res^) expression constructs harboring loss-of-function mutations within the DEXDc- (K785A) and bromodomains (N1540A)^41,42^ (Fig. 3A). Wild-type and mutant forms of SMARCA4^res^ were stably transduced in KYSE-510 cells and SMARCA4^res^ expression was monitored by capillary Western immunoassay. Knock-down of SMARCA4 by siRNA led to depletion of SMARCA4 in parental KYSE-510 cells, but not in KYSE-510 cells expressing SMARCA4^res^ variants (Fig. 3B). In line with previous results (Fig. 2A and Supplementary Fig. S2B), SMARCA4 sgRNAs were strongly depleted in empty vector control-transduced KYSE-510. In contrast, wild-type SMARCA4^res^-expressing KYSE-510 cells were largely rendered inert to loss of endogenous SMARCA4. While ectopic expression of bromodomain-mutant (N1540A) SMARCA4^res^ also protected KYSE-510 cells from SMARCA4 knock-out, the ATP-binding deficient form (K785A) of SMARCA4^res^ did not rescue the depletion of SMARCA4 sgRNA-expressing KYSE-510 cells (Fig. 3C).

Wild-type and mutant forms of SMARCA4^res^ were also stably expressed in the SMARCA2-deficient cell line KYSE-30 (Supplementary Fig. S3A). Similar to KYSE-510 cells, we found that ectopic expression of wild-type or bromodomain-mutant SMARCA4^res^, but not the ATP-binding deficient variant, rescued cells from loss of endogenous SMARCA4 (Supplementary Fig. S3B).

These studies suggest that the dependence of SMARCA2-deficient ESCC cell lines on SMARCA4 is related to SMARCA4 ATP-binding activity.

### SMARCA2 displays an acute and strong synthetic lethal interaction with SMARCA4

**Figure 4:**
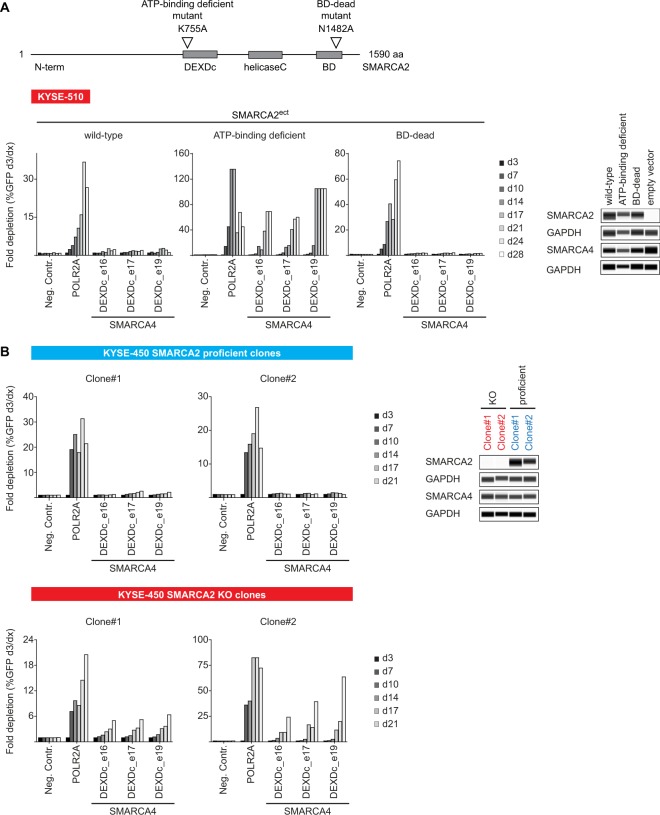
SMARCA2 and SMARCA4 display an acute synthetic lethal interaction in ESCC cells lines. (**A**) SMARCA2 reconstitution in SMARCA2-deficient KYSE-510 cells alleviates SMARCA4 dependency. Location of ATP-binding- and bromodomain (BD)-inactivating mutations in SMARCA2 (designated as SMARCA2^ect^) is indicated. DEXDc, DEAD-like helicases superfamily; BD, bromodomain. Time-resolved CRISPR-Cas9 depletion studies in SMARCA2^ect^ expressing KYSE-510 cells. Fold depletion of sgRNA- and GFP-co-expressing cells relative to day 3 (d3) post-transduction was assessed by flow cytometry analysis over 28 days (n = 1 experimental replicate). Expression of SMARCA2^ect^ variants was determined using capillary Western immunoassay. GAPDH expression was used to monitor equal loading. (**B**) SMARCA2 inactivation elicits dependency on SMARCA4. Time-resolved CRISPR-Cas9 depletion studies in SMARCA2-proficient and -deficient (KO) KYSE-450 monoclonal cell lines. Fold depletion of sgRNA- and GFP-co-expressing cells relative to day 3 (d3) post-transduction was assessed by flow cytometry analysis over 28 days (n = 1 experimental replicate). Knock-out of SMARCA2 was confirmed using capillary Western immunoassay. GAPDH expression was used to monitor equal loading.

To interrogate the synthetic lethal interaction of SMARCA2 and SMARCA4 in ESCC, we ectopically expressed wild-type, DEXDc- (K755A) and bromodomain-mutant (N1482A) forms of SMARCA2 (SMARCA2^ect^) in KYSE-510 cells lacking endogenous SMARCA2 expression (Fig. 4A). Introduction of wild-type SMARCA2^ect^ reversed the strong depletion effect of SMARCA4 sgRNAs observed in empty vector control-transduced KYSE-510 cells (Figs 3C and 4A). In agreement with the dependence of KYSE-510 cells on SMARCA4 ATPase, but not bromodomain function (Fig. 3C), bromodomain-mutant SMARCA2^ect^, but not the ATP-binding deficient variant rendered KYSE-510 cells inert to loss of SMARCA4 (Fig. 4A).

To assess whether acute loss of SMARCA2 renders ESCC cell lines vulnerable to SMARCA4 depletion, we generated SMARCA2-null KYSE-450 monoclonal lines using CRISPR-Cas9 mediated gene knock-out (Fig. 4B). No depletion of SMARCA4 sgRNAs was observed in unedited, SMARCA2-proficient KYSE-450 monoclonal lines (Fig. 4B), in line with previous results (Fig. 2A). In contrast, in two individual KYSE-450 SMARCA2 knock-out clones, depletion of SMARCA4-targeting sgRNAs was observed, albeit more modestly than observed for the positive control sgRNA POLR2A (Fig. 4B). We subsequently confirmed these findings with an additional ESCC model, KYSE-150 (Supplementary Fig. S4). Strong depletion of SMARCA4 sgRNAs was not observed in SMARCA2-proficient clones, however, similar to KYSE-450 cells, loss of SMARCA2 rendered KYSE-150 cells dependent on SMARCA4 (Supplementary Fig. S4).

We wanted to extend our studies beyond ESCC cell models. Therefore, we selected non-ESCC cell lines with low SMARCA2 mRNA expression^43^ and identified four cell lines - HCT 116, SK-CO-1 (both colorectal carcinoma), HuP-T4 (pancreas carcinoma) and OV-90 (ovarian carcinoma) - with low or non-detectable SMARCA2 protein expression suitable for CRISPR-Cas9 depletion studies (Supplementary Fig. S5). Similar to the effects observed in ESCC cell lines, strong depletion of SMARCA4 sgRNAs was observed in all four SMARCA2-deficient cell models tested.

In summary, the SMARCA2 reconstitution and knock-out studies demonstrate a strong and hard-wired synthetic lethal interaction of SMARCA2 and SMARCA4 in ESCC cell models. Further, our data highlight SMARCA4 as a universal vulnerability of SMARCA2-deficient cancer cells, irrespective of the tumor type.

### Pharmacological targeting of SMARCA4 impairs viability of a SMARCA2-null ESCC model

**Figure 5:**
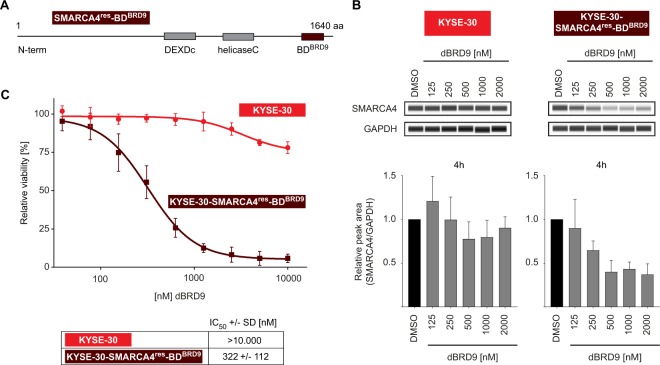
Pharmacological targeting of SMARCA4 impairs proliferation of SMARCA2-deficient ESCC KYSE-30 cells. (**A**) Schematic representation of siRNA/sgRNA-resistant SMARCA4 variant with substituted BRD9 bromodomain (SMARCA4^res^-BD^BRD9^). KYSE-30 cells were stably transduced with SMARCA4^res^-BD^BRD9^ followed by knock-out of endogenous SMARCA4 and generation of a monoclonal cell line (KYSE-30-SMARCA4^res^-BD^BRD9^). (**B**) Capillary Western immunoassay for SMARCA4 in parental KYSE-30 and KYSE-30-SMARCA4^res^-BD^BRD9^ upon dBRD9 treatment for 4 h with the indicated doses. GAPDH expression was used to monitor equal loading. Quantification of SMARCA4 expression (lower panel) is represented as mean ± SD of four independent experiments. (**C**) Cell viability assay. Parental KYSE-30 cells and KYSE-30-SMARCA4^res^-BD^BRD9^ were treated as indicated with dBRD9 and cell viability was determined ten days post treatment. Data are represented as mean ± SD of four independent experiments.

A sgRNA-resistant SMARCA4 expression construct in which the SMARCA4 bromodomain is replaced with the bromodomain of BRD9 (SMARCA4^res^-BD^BRD9^) was designed to determine whether acute degradation of SMARCA4 represents a therapeutic approach against SMARCA2-deficient cancers (Fig. 5A). The bromodomain substitution renders the SMARCA4 variant susceptible to targeted degradation via a potent and highly selective Proteolysis Targeting Chimera (PROTAC) directed against the bromodomain of BRD9, referred to as dBRD9^44^. SMARCA4^res^-BD^BRD9^ was transduced in SMARCA4-dependent KYSE-30 cells and, subsequently, a monoclonal line harboring knock-out of the endogenous form of SMARCA4 was generated (KYSE-30-SMARCA4^res^-BD^BRD9^). Treatment with dBRD9 for four hours led to a decrease in SMARCA4 protein levels in the KYSE-30-SMARCA4^res^-BD^BRD9^ cell line, but not parental KYSE-30 cells (Fig. 5B). Ectopic expression of SMARCA4^res^-BD^BRD9^ rendered KYSE-30-SMARCA4^res^-BD^BRD9^ cells inert to SMARCA4-targeting sgRNAs, indicating functionality and sufficient expression of the bromodomain substituted SMARCA4 variant (Supplementary Fig. S6). In line with the genetic depletion of SMARCA4, dBRD9-mediated degradation of SMARCA4 was accompanied by decreased viability of KYSE-30-SMARCA4^res^-BD^BRD9^ cells with a half maximal inhibitory concentration (IC_50_) of 322 ± 122 nM. In contrast, parental KYSE-30 cells were not affected by treatment with dBRD9 (Fig. 5C).

The results indicate that pharmacological targeting of SMARCA4 might constitute a novel therapeutic approach for the treatment of SMARCA2-deficient tumors.

## Discussion

Besides surgery, chemotherapy and radiotherapy regimens constitute the mainstay of clinical therapy of ESCC. Responses to combination chemotherapy regimens are short-lived in advanced ESCC due to the rapid emergence of resistance^45,46^. While recent large scale genomic analyses have provided a detailed landscape of genomic alterations in ESCC^33–36^, approaches to clinically exploit selective vulnerabilities in ESCC with targeted agents have proven unsuccessful to date^45,47,48^.

The results of this study uncover the BAF complex ATPase SMARCA4 as a novel vulnerability in a subset of ESCC cell lines characterized by low or absent expression of SMARCA2 - a relationship reciprocal to the known synthetic lethal interaction between SMARCA2 and SMARCA4 in SMARCA4-deficient cancer cell lines^25–27^. We demonstrate that lack of SMARCA2 is both required and sufficient to induce dependency on the paralogous ATPase SMARCA4. These findings extend the concept of synthetic lethality between SMARCA2 and SMARCA4 in cancer cells. In addition to ESCC cell models, SMARCA2-deficient colorectal, pancreas and ovarian carcinoma cell lines display SMARCA4 dependency, indicating that the interdependence of SMARCA2 and SMARCA4 is hard-wired and context-independent. SMARCA4 dependency of SMARCA2-deficient cell lines is linked to its ATPase, but not bromodomain, function. This result is in line with a strict requirement on SMARCA2 ATPase activity in SMARCA4-mutant NSCLC cell lines^25,41^. Either SMARCA2 or SMARCA4 are sufficient to rescue from loss of SMARCA2 ATPase activity, further emphasizing the context-independent nature of SMARCA2/‌SMARCA4 synthetic lethality.

Of note, two ESCC cell lines, T.T and KYSE-410, display dependence on SMARCA4 despite readily detectable levels of SMARCA2. In various cellular contexts, SMARCA4 has been linked to an oncogenic function^49^ and high expression levels of SMARCA4 are associated with poor prognosis in multiple tumor types^50^. A critical requirement on SMARCA4 in promoting MYC-dependent transcription has been described in SMARCA2-proficient acute leukemia cell models^51^. Thus, the dependency on SMARCA4 in the presence of SMARCA2 in T.T and KYSE-410 cells might be attributed to a context-dependent requirement on SMARCA4 function distinct from the paralog interdependence. Further work is required to elucidate the mechanisms underlying the dependency on SMARCA4 in the SMARCA2-proficient ESCC cell models.

In contrast, the function of SMARCA4 in cancer has also been linked to a tumor suppressive role. Inactivating mutations of SMARCA4 are frequently observed in human cancer, most prominently in non-small cell lung cancer (NSCLC)^13,22,52–55^. The tumor suppressive function of SMARCA4 is corroborated by *Smarca4* knock-out studies in mice. While homozygous deletion of *Smarca4* is embryonic lethal, heterozygous loss of *Smarca4* predisposes to tumor formation of epithelial origin^56,57^. Lung-specific, conditional ablation of *Smarca4* enhances tumor formation in a carcinogen-induced lung cancer model^58^. Tumor formation in *Smarca4* heterozygous mice occurs with long latency and low penetrance^56,57^, indicating that for treatment of SMARCA4-dependent cancers a therapeutic index for transient inhibition of SMARCA4 might exist without the risk of secondary malignancies.

In contrast, loss-of-function mutations of SMARCA2 are primarily linked to developmental/neurological disorders^59^. Missense mutations or in-frame deletions affecting the ATPase function of SMARCA2 are causative for Nicolaides-Baraitser syndrome, a rare condition characterized by sparse hair, facial and limb abnormalities and intellectual disabilities^60,61^. In cancer, mutations of SMARCA2 are rarely observed^14^. The lack of SMARCA2 expression in cancer cells might therefore be related to cell-specific and epigenetic regulation mechanisms^50,62^. A notable exception is adenoid cystic carcinoma (ACC), a rare type of cancer arising from salivary glands where mutations within the ATPase domain of SMARCA2 are found in approximately 5% of cases^63^, indicating a potential tumor suppressive function of SMARCA2 in this context.

Using a PROTAC approach in an engineered cell model, we demonstrate that pharmacological targeting of SMARCA4 effectively impairs proliferation of a SMARCA2-deficient cell line. Our data suggest that SMARCA4 inhibition would allow for a selective, cancer-cell directed therapy sparing normal, SMARCA2-expressing cells and tissues. Expression of SMARCA2 is predominantly detected in brain, liver, muscle and endothelial cells and has been linked to slow-cycling and differentiated cell states^64^. While it is difficult to define a threshold for expression of SMARCA2 that maintains sufficient BAF functionality in the absence of SMARCA4, the fact that homozygous loss of SMARCA4 is embryonic lethal, indicates that SMARCA2 cannot universally compensate for SMARCA4 function^56^. In adult tissues, dual inactivation of SMARCA2 and SMARCA4 primarily affects viability of vascular endothelial cells^65^. Pharmacological targeting of SMARCA4 would therefore require a thorough analysis of potential toxicities in normal cell and tissues.

In human cancer also concomitant loss of SMARCA2 and SMARCA4 expression has been described^25,66–68^. In small-cell carcinomas of the ovary, hypercalcemic type (SCCOHTs), characterized by bi-allelic inactivating mutations of SMARCA4, loss of SMARCA2 expression is frequently observed^68–71^. These examples depict a striking exemption of the concept of hard-wired SMARCA2/SMARCA4 paralog dependency and constitute a potential mechanism for resistance to BAF targeted cancer therapies^72^. Further studies are required to decipher whether alternative ATP-dependent nucleosome-remodeling complexes, such as ISWI and CHD complexes^73^, can fulfill a compensatory function in the absence of BAF activity.

Our study highlights the use of functional genomic screening for identification of novel vulnerabilities in cancer cell lines. Given the possibility to develop selective SMARCA4 ATPase inhibitors or PROTACs selectively inducing SMARCA4 proteolysis^72,74,75^ and tolerability of systemic SMARCA4 inactivation, the results of this study suggest that targeting of SMARCA4 might be a valuable strategy for the treatment of tumors characterized by low or absent expression of SMARCA2.

## Methods

### Cell culture and lentiviral transduction

KYSE-30 and KYSE-450 (ESCC) were cultured in 45% RPMI 1640 (Gibco) + 45% Ham’s F12 + 10% fetal calf serum (FCS). For cultivation of KYSE-70 and KYSE-410 (ESCC) RPMI 1640 (Gibco) supplied with 10% FCS, whereas for KYSE-140, KYSE-150, KYSE-180, KYSE-510 and COLO-680N (ESCC) RPMI1640 (ATCC #30-2001) + 10% FCS was used. T.T (ESCC) cell line was cultured in DMEM:F12 (ATCC: 30-2006) containing 10% FCS. KYSE-270 (ESCC) was cultured in RPMI1640 (Gibco) + HAM’s F12 (Gibco, 31765-027) (1:1) including 2 mM Glutamine and 2% FCS. HCT 116 (colon carcinoma) cells were cultured in McCoy’s 5 A medium (Gibco, 36600-021) supplemented with GlutaMAX (Thermo, 35050061) and 10% FCS, SK-CO-1 (colon carcinoma) was cultured in EMEM (SIGMA, M5650) with GlutaMAX and 10% FCS supplemented with Sodium-Pyruvate, and OV-90 (ovarian carcinoma) was cultured in a 1:1 mixture of MCDB 105 medium (Sigma, M6395) with glutamine and HEPES (Thermo, 15630080) containing a final concentration of 1.5 g/l sodium bicarbonate and Medium 199 (Sigma, M4530) containing a final concentration of 2.2 g/l sodium bicarbonate. HuP-T4 (pancreas carcinoma) was cultured in MEM + Earl´s Salt (Gibco, 21090-022, no glutamine) + 20% FCS and GlutaMAX.

Lentiviral particles were generated via usage of the Lenti-X Single Shot protocol (Clontech, Mountain View, CA, US). Low passage cell lines were transduced with lentiviral expression vector encoding Cas9 and puromycin and selected over two weeks. Cas9 editing efficacy was monitored via depletion of sgRNAs targeting positive control genes (*CDK1*, *POLR2A* or *RPA3*). For Cas9-expressing cell lines the following concentrations of Puromycin were added to the standard medium: T.T, and KYSE-270: 4 µg/ml; KYSE-70, KYSE-140, HCT 116, and HuP-T4: 2 µg/ml; KYSE-450, KYSE-510, SK-CO-1, and OV-90: 1 µg/ml; COLO-680N: 0.5 µg/ml; KYSE-30, KYSE-150, and KYSE-410: 0.25 µg/ml. The medium for the cell lines expressing SMARCA4^res^, SMARCA2^ect^, or SMARCA4-BD^BRD9^ was supplied with 25 µg/ml Hygromycin B. All supplements were obtained from Gibco, FCS (SH30071.03) from GE Healthcare Life Sciences, Puromycin from Sigma (P9620), and Hygromycin B from Invitrogen (10687010). Sources and information for authentication of cell lines (STR fingerprinting at Eurofins Genomics, Germany) used in this study are attached in Supplementary Table 1. All cell lines were tested negatively for mycoplasma contamination.

### CRISPR-Cas9 epigenome sgRNA library screens

Cas9-expressing ESCC cell lines were transduced with the indicated sgRNA library (Supplementary Dataset File 1) at a multiplicity of infection of approximately 0.3 and cultured for 18 population doublings. Genomic DNA was purified using QIAamp DNA MiniKit (50) (Qiagen, 51304) and amplicons around the sgRNA sequences were PCR amplified using the following primers: LRG_F2: TCTTGTGGAAAGGACGAAACACCG; LRG_R2: TCTACTATTCTTTCCCCTGCACTGT. The PCR product of 40 PCR reactions was pooled and purified using QIAquick PCR Purification Kit (250) (Qiagen, 28106) and 50 ng of amplicons were used for the library generation with the TruSeq Nano DNA Library Prep kit for NeoPrep (Illumina). The sequencing was conducted on a HiSeq. 1500 (Illumina) in rapid mode with the paired end protocol for 50 cycles.

### Bioinformatics analysis of CRISPR-Cas9 screens

Statistical analysis of depletion signals was performed with the MAGeCK tool (V 0.5.6)^76,77^. First guide level counts were generated from sequencing data with the ‘mageck count’ function with parameter ‘-norm-method control’. The set of negative control guides was derived from genes that never show strong depletion signals in the AVANA data set^40^ and that overlap with genes in our library. Next the ‘mageck test’ function was run with parameters ‘-remove-zero none -gene-lfc-method median’ to derive gene-level α-RRA scores for each cell line. To improve the comparability between the cell lines, α-RRA scores were scaled by using three positive control genes (*CDK1*, *POLR2A*, *RPA3*) such that the mean of these control genes was −1 for all cell lines.

### CRISPR singleton-gRNA depletion experiments

The sgRNAs were designed using a tool provided by Zhang *et al*. (http://crispr.mit.edu/) and cloned into GFP encoding vectors. Cas9-expressing cells were transduced and the fraction of GFP-positive cells was determined on day 3 post-transduction. Cells were split for 21-28 days. Fold changes of initially transduced cell populations were calculated from day 3 to the respective time points. For the heatmap representation, depletion of GFP-positive cell populations on day 28 was related to the fraction  of GFP-positive cells  of the POLR2A positive control on day 28.

### Sequences

All sgRNA sequences used for depletion experiments and generation of knock-out cell lines are listed in Supplementary Dataset File 1.

### Quantitative reverse transcription PCR (qRT-PCR)

RNA was isolated using RNeasy Mini Kit (Qiagen, 74106) and reversly transcribed using SuperScript^™^ VILO^™^ kit (Thermo Scientific). For qPCR analysis, QuantiTect® Multiplex PCR kit (Qiagen, Hilden, Germany) and StepOne Real-Time PCR Sytem™ (Applied Biosytems) was used. The respective primers were all ordered from Applied Biosystems, including house-keeping genes: 18 S rRNA (VIC®/MGB, 4319413E), ACTB (VIC®/MGB, 4326315E), GAPDH (VIC®/MGB, 4326317E) and primers for SMARCA2 (Hs01030858_m1 MGB/FAM) and SMARCA4 (Hs00231324 MGB/FAM). SMARCA2/4 expression was calculated from duplicates in relation to three different house-keeping genes listed above.

### Capillary western immunoassay

Lysates were generated using MSD Tris Lysis Buffer (Mesoscale #R60TX-2) supplied with Protease & Phosphatase Inhibitor Cocktail (1:100, Thermo Scientific#815-968-0747). Capillary Western immunoassay (Separation module, SMW004-1) was conducted according to the manufacturer’s protocol. Dilutions of protein were prepared to obtain a final protein concentration of 0.4 µg/µl.

### Antibodies

Antibodies used were anti-BRG1/SMARCA4 (Cell Signaling #49360, 1:20 dilution for capillary Western immunoassay), anti-SMARCA2 (SIGMA #HPA029981, 1:20 dilution for capillary Western immunoassay) and anti-GAPDH (abcam #ab9485, 1:10000 dilution for capillary Western immunoassay). Respective antibodies were used in a multiplex assay together with anti-GAPDH for relative quantification of protein levels.

### Correlation of SMARCA4 dependency and SMARCA2 expression

SMARCA2 gene expression values (TPM – transcripts per million) were obtained from https://ordino.caleydoapp.org/ ^43^. SMARCA4 sensitivity measurements were obtained from McDonald *et al*.^39^ (RSA scores) and Meyers *et al*.^40^ (Ceres scores). The visualizations and statistical tests were performed using R version 3.5.0 (R Core Team (2018) R: A language and environment for statistical computing, R Foundation for Statistical Computing, Vienna, Austria; available online at https://www.R-project.org/.), including the R package ggplot2 version 3.0.0 (H. Wickham (2016) ggplot2: Elegant Graphics for Data Analysis; 10.1007/978-3-319-24277-4).

### cDNA transgene vectors

For expression of SMARCA4^res^ and SMARCA2^ect^ the following constructs were generated by gene synthesis (GenScript, China) based on the SMARCA4 cDNA sequence NCBI NM_ 001128844.1 and SMARCA2 cDNA sequence NCBI NM_003070.5 and cloning into the parental pLVX vector (Clontech, Mountain View, CA, US): pLVX-empty-IRES-Hygro; pLVX-SMARCA4-wt-IRES-Hygro; pLVX-SMARCA4-ATP-binding deficient (K785A)-IRES-Hygro; pLVX-SMARCA4-BD-dead(N1540A)-IRES-Hygro; pLVX-SMARCA2-wt-IRES-Hygro; pLVX-SMARCA2-ATP-binding-deficient(K755A)-IRES-Hygro; pLVX-SMARCA2-BD-dead(N1482A)-IRES-Hygro; pLVX-SMARCA4^res^-BD^BRD9^-IRES-Hygro. ATP-binding deficient and BD-dead sites were selected similar to known inactivating mutations in SMARCA2/4^41,42^. The sequence of SMARCA4^res^-BD^BRD9^ is shown in Supplementary Table 3. All constructs were codon optimized to render the transgenes resistant towards siRNAs and sgRNAs. For SMARCA4, DEXDc (DEAD-like helicases superfamily) and HELIC (helicaseC) domains were annotated according to UniProt entry P51532, bromodomain (BD) was annotated according to NCBI entry 6597 (cd05516). For SMARCA2, domains were annotated according to UniProt entry P51531, bromodomain (BD) was annotated according to NCBI entry 6595 (cd05516).

### siRNA transfection

For siRNA-mediated gene knock-down, cells were reversely transfected using Lipofectamine RNAiMAX reagent according to the manufacturer’s protocol (Invitrogen, Waltham, MA, US). The final concentration of the respective siRNAs was 25 nM. The siRNA duplexes were purchased from Thermo Scientific/ Dharmacon: Non-targeting pool (D-001810-10-20), SMARCA4_1 (J-010431-06), SMARCA4_2 (J-010431-07).

### CRISPR-Cas9-mediated gene knock-out

All-in-one pSpCas9 BB-2A-GFP (PX458) vectors were ordered from Genscript, China with sgRNAs targeting the N-terminus of SMARCA2/4 as listed in Supplementary Table 2. Cells were transfected using Lipofectamine 3000 reagent (Thermo Fisher Scientific, L3000015) and GFP-positive cells were sorted after 48 h using flow cytometry (SH800S Cell Sorter,Sony Biotechnology) and diluted to obtain single cell clones. Knock-out was monitored using capillary Western immunoassay.

### Cell viability assay

KYSE-30-SMARCA4^res^-BD^BRD9^ and parental KYSE-30 cells were cultured for approximately 18 passages post-thawing and plated at low densities (100 cells/well) in a 96-well plate. The next day, cells were treated with varying doses of dBRD9^44^ as indicated. Viability was determined using CellTiter-Glo (Promega, Madison, WI, US). Ten days post-treatment, 100 μl CellTiter-Glo solution were added directly to the cell medium, mixed and incubated for 10 min prior to determination of the luminescence signal.

## Supplementary information

Supplementary Information

Dataset 1

Dataset 2

Dataset 3

## Data Availability

All data generated or analyzed during this study are included in this published article and its Supplementary Information files.
