# Supplementary material for: SMARCA2-deficiency confers sensitivity to targeted inhibition of SMARCA4 in esophageal squamous cell carcinoma cell lines
Source: Sci Rep. 2019 Aug 12;9:11661. doi: 10.1038/s41598-019-48152-x (PMC6691015; doi:10.1038/s41598-019-48152-x)
Supplement: Supplementary file 1 — Supplementary Information [file 41598_2019_48152_MOESM1_ESM.pdf]

SMARCA2-deficiency confers sensitivity to targeted inhibition of SMARCA4 in esophageal squamous cell carcinoma cell lines

Katharina Ehrenhöfer-Wölfer[1], Teresa Puchner[1], Cornelia Schwarz[1], Janine Rippka[1], Silvia Blaha-Ostermann[1], Ursula Strobl[1], Alexandra Hörmann[1], Gerd Bader[1], Stefan Kornigg[1], Stephan Zahn[1], Wolfgang Sommergruber[1], Norbert Schweifer[1], Thomas Zichner[1], Andreas Schlattl[1], Ralph A. Neumüller[1], Junwei Shi[2], Christopher R. Vakoc[3], Manfred Kögl[1], Mark Petronczki[1], Norbert Kraut[1], Mark A. Pearson[1] and Simon Wöhrle[1]*

[1]Boehringer Ingelheim RCV GmbH & Co KG, 1120 Vienna, Austria

[2]Department of Cancer Biology, University of Pennsylvania, Philadelphia, PA 19104, USA

[3]Cold Spring Harbor Laboratory, Cold Spring Harbor, NY 11724, USA

*Corresponding author

simon.woehrle@boehringer-ingelheim.com

Supplementary Figure S1 – SMARCA4 dependency anti-correlates with SMARCA2 expression

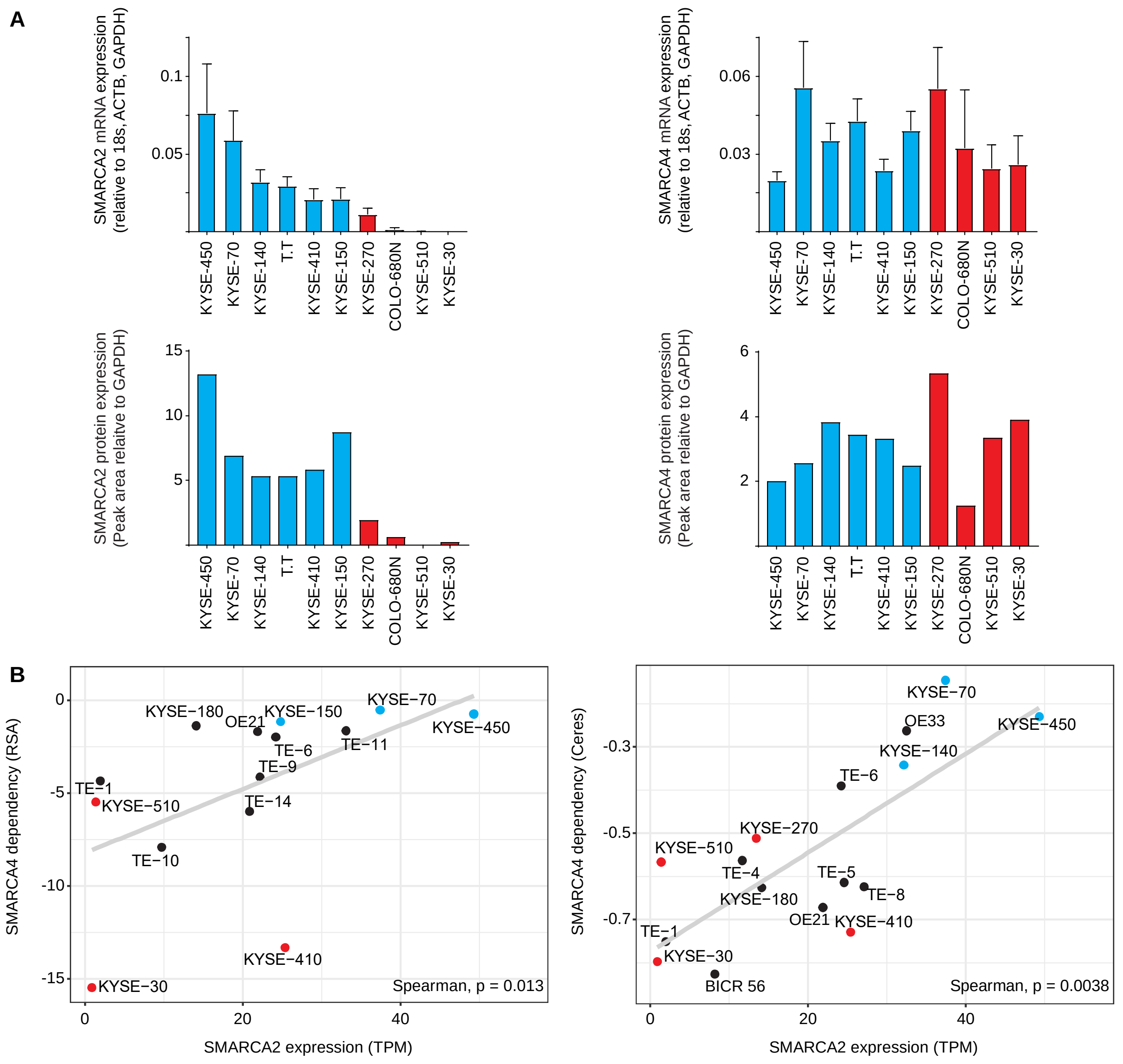

A – Determination of SMARCA2 and SMARCA4 expression levels via qRT-PCR (upper panels) and capillary Western immunoassay (lower panels) in the ESCC cell lines used for CRISPR-Cas9 depletion screens. For qRT-PCR SMARCA2 or SMARCA4 expression was normalized to three different house-keeping genes (*18s rRNA, ACTB, GAPDH*). qRT-PCR data are represented as mean ± SD of three independent experiments. SMARCA2 or SMARCA4 protein levels are normalized to GAPDH expression (n=1 experimental replicate).

B – Correlation of SMARCA4 dependency scores in ESCC cell models derived from McDonald et al. (RSA scores, left panel) and Meyers et al. (Ceres scores, right panel) [39,40] with SMARCA2 gene expression levels (transcripts per million, TPM) [43]. Cell lines used in CRISPR-Cas9 depletion screens are indicated either in red (SMARCA2-deficient) or blue

(SMARCA2-proficient). Low RSA and Ceres values indicate SMARCA4 dependency. Spearman correlation was used for statistical analysis: SMARCA4 (RSA):SMARCA2 (TPM) $p=0.013$; SMARCA4 (Ceres):SMARCA2 (TPM) $p=0.0038$.

Supplementary Figure S2 – Selection of most efficient sgRNAs using singleton-depletion experiments

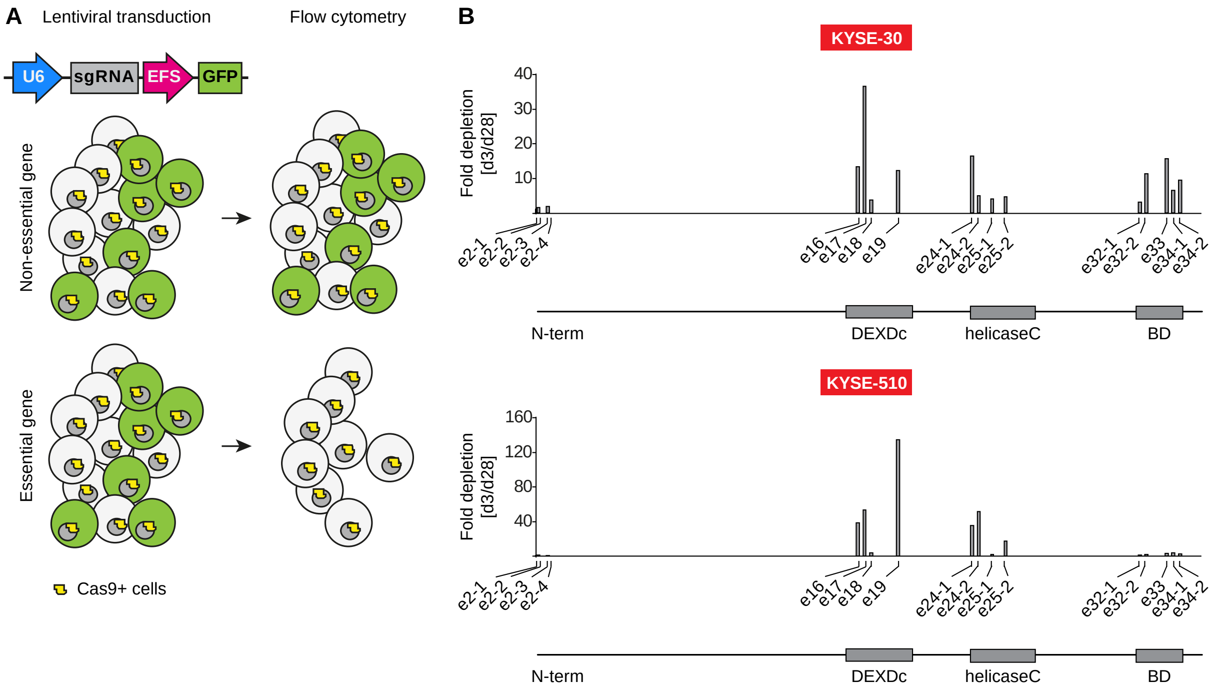

A – Scheme of sgRNA-depletion experiments using individual sgRNAs and flow cytometry analysis of sgRNA- and GFP-co-expressing cells. Cas9 expressing cells were transduced with constructs encoding sgRNA and GFP at a multiplicity of infection of approximately 0.3. The fraction of GFP-expressing cells was assessed over time by flow cytometry. Cells expressing sgRNAs targeting cell essential genes are depleted from the population, while the fraction of cells harboring sgRNAs targeting non-essential genes remains constant.

B – SMARCA4 CRISPR scanning in KYSE-30 and KYSE-510 ESCC cell lines. Cas9 expressing KYSE-30 and KYSE-510 cell lines were transduced with a lentivirus encoding GFP and sgRNAs targeting multiple domains in SMARCA4 as indicated and fold depletion of sgRNA- and GFP-co-expressing cells relative to day 3 (d3) post-transduction was assessed by flow cytometry on day 28 (d28) (n=1 experimental replicate). N-term, N-terminal encoding region; DEXDc, DEAD-like helicases superfamily; BD, bromodomain.

Supplementary Figure S3 – SMARCA4 ATPase domain is the functional important domain

A

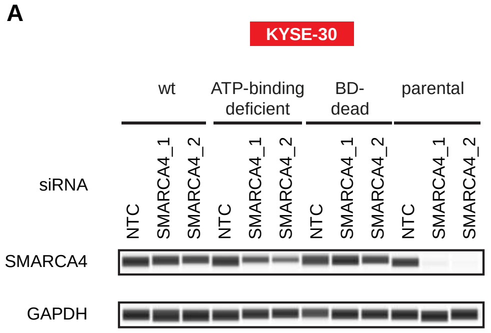

B

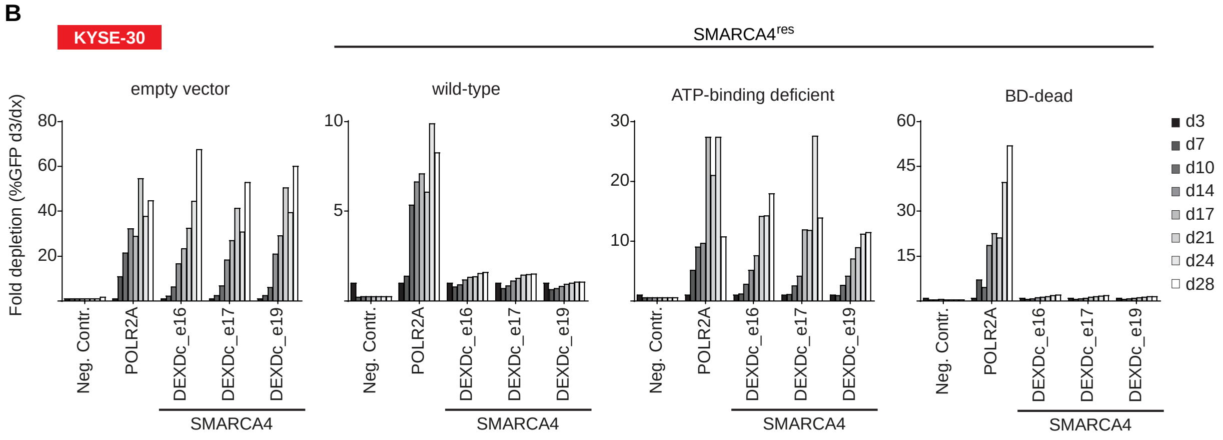

A – SMARCA2-deficient KYSE-30 cells were stably transduced with wild-type or mutant forms of SMARCA4^{res}. Transgene expression was monitored by capillary Western immunoassay in the presence of siRNA-mediated knock-down of endogenous SMARCA4. Lysates were prepared 72 h post siRNA transfection. GAPDH expression was used to monitor equal loading.

B – Time-resolved CRISPR-Cas9 depletion studies in SMARCA4^{res} expressing KYSE-30 cells. Fold depletion of sgRNA- and GFP-co-expressing cells relative to day 3 (d3) post-transduction was assessed by flow cytometry analysis over 28 days (n=1 experimental replicate).

Supplementary Figure S4 – SMARCA2 inactivation elicits dependency on SMARCA4

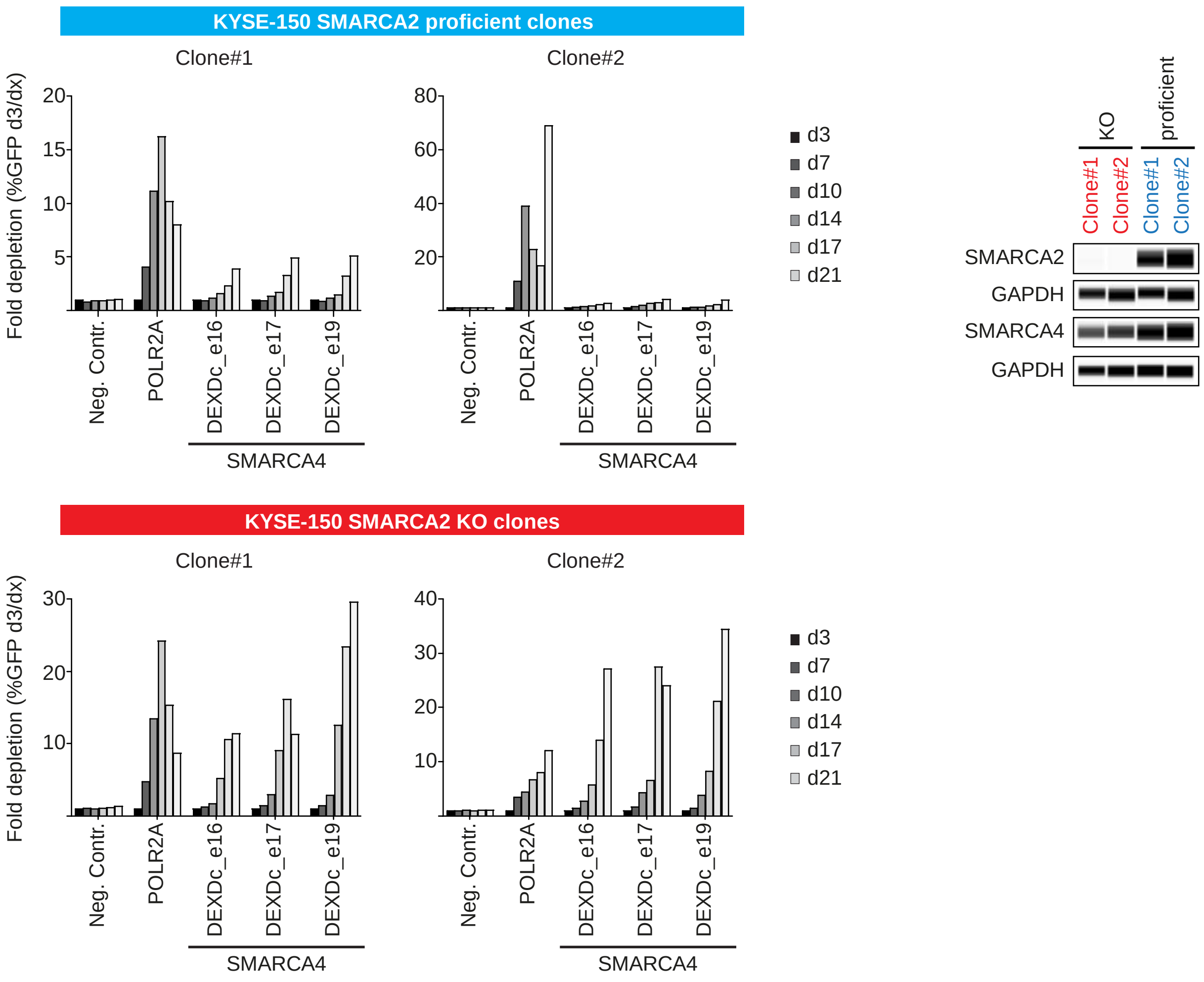

Time-resolved CRISPR-Cas9 depletion studies in SMARCA2-proficient and -deficient (KO) KYSE-150 monoclonal cell lines. Fold depletion of sgRNA- and GFP-co-expressing cells relative to day 3 (d3) post-transduction was assessed by flow cytometry analysis over 21 days (n=1 experimental replicate). Knock-out of SMARCA2 was confirmed using capillary Western immunoassay. GAPDH expression was used to monitor equal loading.

Supplementary Figure S5 – SMARCA2-deficient colon, ovarian and pancreas carcinoma cell lines are SMARCA4-dependent

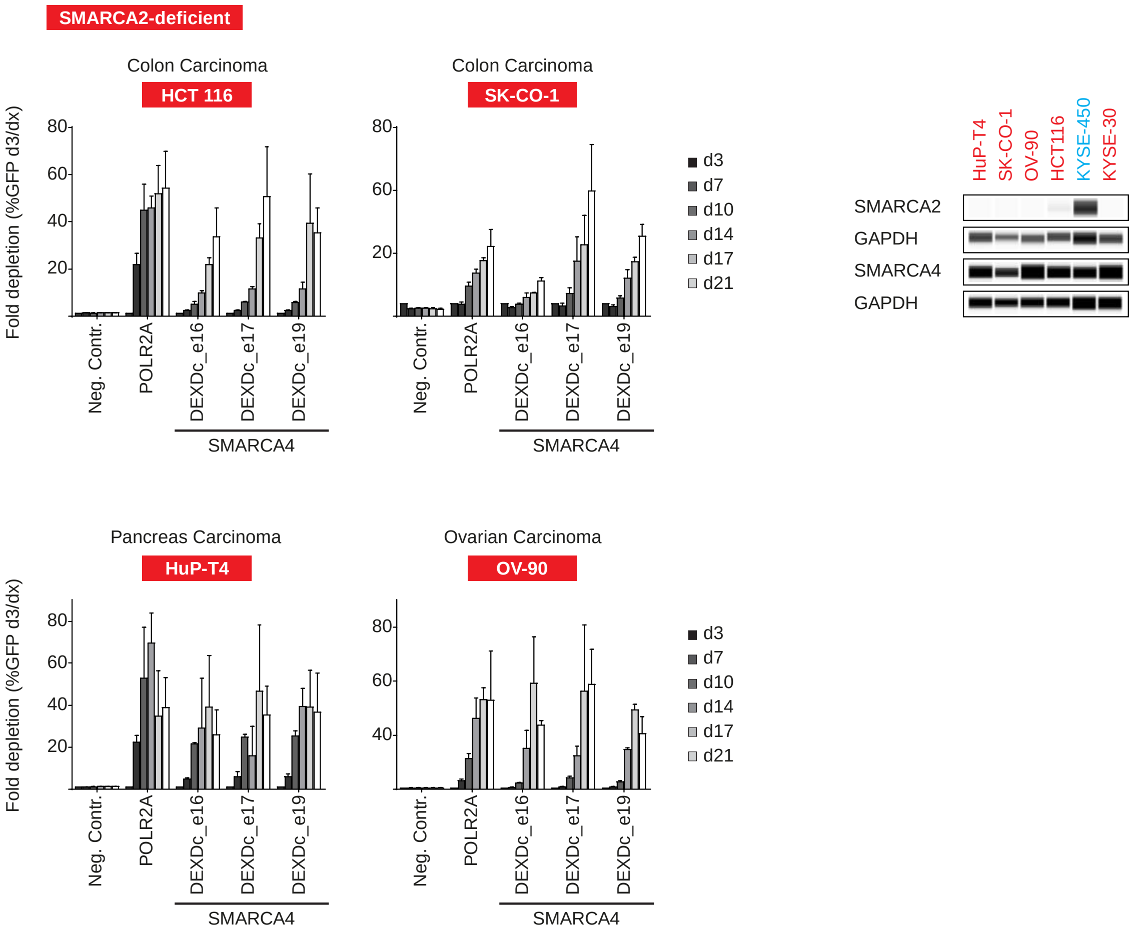

Time-resolved CRISPR-Cas9 depletion studies in SMARCA2-deficient HCT 116, SK-CO-1 (colon carcinoma), OV-90 (ovarian carcinoma) and HuP-T4 (pancreas carcinoma) cell lines. Fold depletion of sgRNA- and GFP-co-expressing cells relative to day 3 (d3) post-transduction was assessed by flow cytometry analysis over 21 days. Data are represented as mean ± SD of three independent experiments. SMARCA2 expression was monitored using capillary Western immunoassay. GAPDH was used to monitor equal loading.

Supplementary Figure S6 – KYSE-30-SMARCA4^{res}-BD^{BRD9} cells are rendered insensitive to sgRNAs targeting SMARCA4

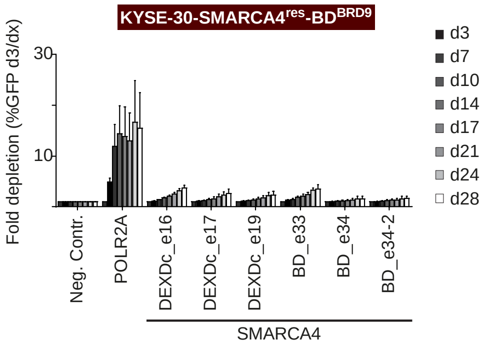

Time-resolved CRISPR-Cas9 depletion studies in Cas9-expressing KYSE-30-SMARCA4^{res}-BD^{BRD9} cells. Fold depletion of sgRNA- and GFP-co-expressing cells relative to day 3 (d3) post-transduction was assessed by flow cytometry analysis over 28 days (n=1 experimental replicate).

Supplementary Table 1 – Overview of cell lines used in this study

Cell line	Tumor type	Source	Source (parental line)	STR confirmed
COLO-680N_Cas9	ESCC	This study	DSMZ (#ACC 182)	Yes
HCT 116_Cas9	CRC	This study	ATCC (#CCL-247)	Yes
HuP-T4_Cas9	Pancreas carcinoma	This study	DSMZ (#ACC 223)	Yes
KYSE-30_Cas9	ESCC	This study	DSMZ (#ACC 351)	Ongoing
KYSE-70_Cas9	ESCC	This study	DSMZ (#ACC 363)	Ongoing
KYSE-140_Cas9	ESCC	This study	DSMZ (#ACC 348)	Ongoing
KYSE-150_Cas9	ESCC	This study	DSMZ (#ACC 375)	Ongoing
KYSE-270_Cas9	ESCC	This study	DSMZ (#ACC 380)	Yes
KYSE-410_Cas9	ESCC	This study	DSMZ (#ACC 381)	Ongoing
KYSE-450_Cas9	ESCC	This study	DSMZ (#ACC 387)	Ongoing
KYSE-510_Cas9	ESCC	This study	DSMZ (#ACC 374)	Ongoing
OV-90_Cas9	Ovarian carcinoma	This study	ATCC (#CRL-11732)	Yes
SK-CO-1_Cas9	CRC	This study	ATCC (#HTB-39)	Yes
T.T_Cas9	ESCC	This study	JCRB (#JCRB0262)	Ongoing

Cell lines used in this study are listed with tumor type of origin and STR authentication information.

Supplementary Table 2 – sgRNAs sequences used in CRISPR-Cas9 depletion assays with individual sgRNAs and for SMARCA2/SMARCA4 gene knock-out

**sgRNAs for depletion experiments**		
**Name**	**Target gene**	**Sequence**
**Control sgRNAs**		
Neg. Contr.	none	GATACACGAAGCATCACTAG
POLR2A	POLR2A	GTACAATGCAGACTTTGACG
**SMARCA4 sgRNAs (N- to C- terminal order)**		
SMARCA4_N-term_e2-1	SMARCA4	TGGCCGAGGAGTTCCGCCCA
SMARCA4_N-term_ e2-2	SMARCA4	CTGGCCGAGGAGTTCCGCCC
SMARCA4_N-term_ e2-3	SMARCA4	GGCCGAGGAGTTCCGCCCAG
SMARCA4_N-term_ e2-4	SMARCA4	CCGGCGAGGGACCCGGGCTA
SMARCA4_DEXDc_e16	SMARCA4	GAGGTACGTGATGAGCGCGA
SMARCA4_DEXDc_e17	SMARCA4	GTCAAACTCGTACGCCCAGT
SMARCA4_DEXDc_e18	SMARCA4	TGAACTTCCCACTCCGGAGC
SMARCA4_DEXDc_e19	SMARCA4	GAACAAGCTTCCCGAGCTCT
SMARCA4_HELIC_e24.1	SMARCA4	GTGGTTGGTTGCTCGGAGTT
SMARCA4_HELIC_e24.2	SMARCA4	GAAGATTACTTTGCGTATCG
SMARCA4_HELIC_e25.1	SMARCA4	CTGAAAACCTTCAACGAGCC
SMARCA4_HELIC_e25.2	SMARCA4	TGATCACAGTGTCTGCCGAC
SMARCA4_BD_e32_109.8	SMARCA4	CTCGGGCAGCTCCTTTCGCG
SMARCA4_BD_e32_109.9	SMARCA4	TCGGGCAGCTCCTTTCGCGA
SMARCA4_BD_e33_102.5	SMARCA4	GGTTGAAGGTCTGTGCGTTC
SMARCA4_BD_e34_59.1	SMARCA4	AGTCGGTCTTCACCAGCGTG
SMARCA4_BD_e34_59.2	SMARCA4	GAAGACCGACTGCAAGACGA

**gRNAs for gene knock-outs**		
SMARCA2_N-term_e2-1	SMARCA2	TCCCATCCTATGCCGACGAT
SMARCA4_N-term_e2-4	SMARCA4	CCGGCGAGGGACCCGGGCTA

Sequences for positive/negative control and SMARCA2/SMARCA4 targeting sgRNAs are indicated. SMARCA4 targeting sgRNAs are listed in N- to C- terminal order according to representation in Supplementary Figure S2. N-term, N-terminal domain; DEXDc, DEAD-like helicases superfamily; HELIC, helicaseC; BD, bromodomain.

Supplementary Table 3 – Substituted amino acids of SMARCA4 and BRD9 bromodomains in the SMARCA4^{res}-BD^{BRD9} variant

**Substituted amino acids of SMARCA4 and BRD9 bromodomains in the SMARCA4^{res}-BD^{BRD9} variant (amino acids representing bromodomains in SMARCA4 (NCBI 6597, cd05516) and BRD9 (UniProt Q9H8M2) are indicated in red font)**	
**SMARCA4**	AEKLSPNPPNLTKKMKKIVDAVIKYKDSSSGRQLSEVFIQLPSRKELPEYYELIRKPVDFKKIKERIRNHKYRSLNDLEKDVMLLCQNAQTFNLEGSLIYEDSIVLQSVFTSVRQKIEKEDD
**BRD9**	AENESTPIQQLLEHFLRQLQRKDPHGFFAFPVTDAIAPGYSMIIKHPMDFGTMKDKIVANEYKSVTEFKADFKLMCDNAMTYNRPDTVYYKLAKKILHAGFKMMSKERLLALKRS

Sequences for SMARCA4 and BRD9 was obtained from NCBI NM_ 001128844 and NM_001317951. Substituted amino acids of bromodomains and flanking regions are indicated. SMARCA4 bromodomain (BD) was annotated according to NCBI entry 6597 (cd05516). BRD9 bromodomain was annotated according to UniProt Q9H8M2.

Supplementary Dataset File Legends

Supplementary Dataset File 1 – List of sgRNAs of the epigenome library

Targeted genes are listed in alphabetic order with indicated sgRNA sequences. For singleton gRNA depletion experiments negative control "neg07" was used. For SMARCA4, DEXDc (DEAD-like helicases superfamily) and HELIC (helicaseC) domains were annotated according to UniProt entry P51532, bromodomain (BD) was annotated according to NCBI entry 6597 (cd05516). For SMARCA2, domains were annotated according to UniProt entry P51531, bromodomain (BD) was annotated according to NCBI entry 6595 (cd05516).

Supplementary Dataset File 2 – Epigenome CRISPR-Cas9 screen results

α-RRA scores for genes included in the epigenome sgRNA library from CRISPR-Cas9 depletion studies in ten ESCC cell lines.

Supplementary Dataset File 3 – Fold change depletion values for individual sgRNAs from epigenome CRISPR-Cas9 screens

Fold change depletion values ($\log_2$) of sgRNA read counts for individual sgRNAs in the epigenome sgRNA library from CRISPR-Cas9 depletion studies in ten ESCC cell lines.
